# The Effect of *G. applanatum* Crude Polysaccharide Extract on Proinflammatory Cytokines and Proapoptotic Caspases in HeLa Cell Line: An *In Vitro* Study

**DOI:** 10.1155/2023/3593295

**Published:** 2023-09-19

**Authors:** Qurrotu A'yun, Raden Joko Kuncoroningrat Susilo, Suhailah Hayaza, Nur'aini Fikriyah, Fina Syifa'una Musthoza, Ufairanisa Islamatasya, Aulia Umi Rohmatika, Dwi Winarni, Sri Puji Astuti Wahyuningsih, Ruey-an Doong, Deya Karsari, Aristika Dinar Yanti, Mochammad Zakki Fahmi, Win Darmanto

**Affiliations:** ^1^Magister Program in Biology, Faculty of Science and Technology, Universitas Airlangga, Surabaya 60115, Indonesia; ^2^Department of Nanotechnology Engineering, Faculty of Advance Technology and Multidiscipline, Universitas Airlangga, Surabaya 60115, Indonesia; ^3^Department of Biology, Faculty of Science and Technology, Universitas Airlangga, Surabaya 60115, Indonesia; ^4^Institute of Analytical and Environmental Sciences, National Tsing Hua University, Sec. 2 Kuang Fu Road, Hsinchu 30013, Taiwan; ^5^Stem Cell Research and Development Center, Universitas Airlangga, Surabaya 60115, Indonesia; ^6^Department of Chemistry, Faculty of Science and Technology, Universitas Airlangga, Surabaya 60115, Indonesia; ^7^Institute of Science Technology and Health, Jombang 61419, Indonesia

## Abstract

Polysaccharide extracts exhibit promise as potential anticancer agents. Among the fungi rich in polysaccharide content, *G. applanatum* stands out; however, its anticancer activity necessitates further investigation. This study aims to explore the impact of *G. applanatum* crude polysaccharide (GACP) extract by assessing its effects on cell viability, levels of proinflammatory cytokines such as TNF-*α*, IFN-*γ*, IL-2, and IL-12, and levels of proapoptotic markers including caspase-3 and caspase-9, as well as the percentages of necrosis and apoptosis in the HeLa cell line. Employing the HeLa cell line as a research model, four groups were studied: KN (media and DMSO), *K*+ (doxorubicin 10 *μ*g/mL), P1 (*G. applanatum* extract 200 *μ*g/mL), and P2 (*G. applanatum* extract 400 *μ*g/mL). The *G. applanatum* extract was obtained via boiling distilled water. Anticancer activity was evaluated through the MTT test (3-(4,5-dimethylthiazole-2-yl)-2,5-diphenyltetrazolium bromide) conducted over three treatment durations (24, 48, and 72 hours). Cytokine levels and caspase-3 and caspase-9 levels were assessed using the ELISA test. Cell apoptosis was determined using the Annexin V-PI biomarker and analyzed through flow cytometry. The MTT test exhibited optimal results at the 48-hour treatment mark. Cytokine level analysis revealed significant reductions in TNF-*α*, IFN-*γ*, IL-2, and IL-12 levels (*p* < 0.005). Concurrently, caspase-3 and caspase-9 levels exhibited substantial increases (*p* < 0.005). Flow cytometry highlighted the highest percentage of apoptosis in HeLa cells. In conclusion, *G. applanatum*'s polysaccharide extract demonstrates potential as an anticancer and therapeutic agent for cancer treatment.

## 1. Introduction

Cancer is the second leading cause of death worldwide [1], with cervical cancer displaying an escalating incidence, particularly prominent in Indonesia. Cervical cancer, primarily induced by human papillomavirus (HPV) infection, notably HPV16 and HPV18 [2–4], is influenced by diverse factors encompassing genetics, dietary habits, oxidative stress, environmental toxins, and psychological stress [5–7]. While early-stage cervical cancer may remain asymptomatic, advanced stages manifest symptoms such as vaginal bleeding, ureteral obstruction, hydronephrosis, uremia, and pericarditis [8–11]. The significance of proinflammatory cytokines lies in their role as stimulators in phosphorylating the inhibitor of *κ*B (IKK) complex, a pivotal step in translocating NF-*κ*B to the nucleus [12–16]. Subsequently, NF-*κ*B engages in transcription, yielding proinflammatory cytokines that foster cancer cell survival. Current treatment modalities for cervical cancer span surgery, chemotherapy, and radiotherapy [17, 18], yet their efficacy is hampered by considerable side effects and susceptibility to relapse [12].

Consequently, the quest for efficacious, affordable, and nontoxic cancer treatments intensifies, fostering interest in exploring natural sources such as *G. applanatum*. This particular fungus features in pharmacological studies, gaining prominence within traditional Chinese medicine due to its attributed medicinal properties spanning anticancer, antidiabetic, antifibrotic, and anti-inflammatory effects [13–16]. *G. applanatum* comprises diverse bioactive constituents, notably polysaccharides and triterpenoids [19–24]. Polysaccharides' biological activity correlates closely with factors such as sulfate content, molecular weight, and glycosidic bond type [25, 26]. Low molecular weight polysaccharides exhibit anticancer potential against various tumor types. Notably, *G. applanatum*'s polysaccharide content demonstrates antimicrobial, antioxidant, anti-inflammatory, and antitumor properties [27–30]. Existing research underscores polysaccharides' role in regulating cell cycle, apoptosis, and autophagy in cancer [31–33]. However, the specific impact of *G. applanatum* polysaccharides on cancer cells through the apoptosis pathway remains an area warranting exploration. This study delves into the influence of *G. applanatum* crude polysaccharide (GACP) extract on proinflammatory cytokines and its induction of apoptosis in HeLa cells.

## 2. Materials and Methods

### 2.1. *G. applanatum* Crude Polysaccharide Extract Preparation

*G. applanatum* specimens were sourced from Tulungagung, East Java, Indonesia. The extraction procedure, as detailed by Susilo et al. [14], was pursued. Dr. Ni'matuzzahroh conducted the identification based on a designated key book [19]. In essence, the basidiocarp of *G. applanatum* was sectioned and air-dried, after which the dried segments were milled into a fine powder. The powdered material was weighed and subjected to boiling at temperatures between 90 and 100°C for a duration of 6 hours. Following the boiling phase, the filtrate extracted from *G. applanatum* underwent centrifugation at 4300 rpm for 5 minutes. The resulting supernatant was precipitated using absolute ethanol in a 1 : 3 ratio. This precipitation process was reiterated three times, and the ensuing pellets were isolated. The pellets were subsequently dissolved in distilled water and again subjected to centrifugation, following the same approach. The resultant pellets were freeze-dried to yield the GACP extract.

### 2.2. FTIR Analysis

For FTIR analysis, the GACP extract was incorporated into potassium bromide (KBr) pellets and assessed using the Nicolet™ iS20 FTIR Spectrometer.

### 2.3. HeLa Cell Culture

HeLa cells were procured from Stem Cell Research and Development at Universitas Airlangga. The cells were cultivated in DMEM and incubated at a temperature of 37°C in an atmosphere containing 5% CO_2_. The administration of GACP extract was undertaken based on specific concentrations allocated to each group, facilitated by a DMSO solvent at levels below 1%. The treatment doses applied in this investigation encompassed the *K*− group (negative control: media + HeLa cell line), *K*+ group (positive control: HeLa cell line + 10 *µ*g/mL doxorubicin), P1 group (HeLa cell line + GACP 200 *µ*g/mL), and P2 group (HeLa cell line + GACP 400 *µ*g/mL). Cells were subsequently cultured for durations of 24, 48, and 72 hours before being harvested to facilitate further assessments.

### 2.4. Cell Viability

The evaluation of HeLa cell viability entailed the utilization of the MTT technique (3-(4,5-dimethylthiazole-2-yl)-2,5-diphenyltetrazolium bromide) [34]. Cells procured from the CO_2_ incubator underwent meticulous examination to confirm their uncontaminated status. For cell harvest, cultures achieving an 80% confluence were selected. Postharvest, cell enumeration, and subsequent dilution with complete culture medium occurred. The cells were then dispensed into a 96-well plate, allotting 5 × 10^3^ cells to each well, followed by overnight incubation. Varied concentrations of GACP extract (200 *µ*g/mL and 400 *µ*g/mL) were introduced, employing DMSO as a cosolvent. This assembly was then incubated in a 37°C, 5% CO_2_ incubator for a duration of 24 hours. At the conclusion of incubation, 100 *μ*L of MTT reagent (0.5 mg/mL) dissolved in DMEM was appended to each well. A subsequent 3-hour incubation at 37°C ensued, resulting in the formation of formazan. The cellular examination was conducted employing an inverted microscope. Subsequent to the distinct appearance of formazan, the introduction of a 10% SDS halt solution in 0.1 N HCl took place. The plate, enveloped in aluminum foil, was relegated to an obscure setting overnight. The optical density (OD) values were gauged via a microplate reader at a wavelength of 595 nm.

### 2.5. Cytokine Levels Analysis

ELISA kits (Bioassay Technology, Shanghai, China) facilitated the quantification of cytokine levels in accordance with the manufacturer's stipulations. Each well of a well plate received a total of 40 L of HeLa cell supernatant. Sequentially, 10 *μ*L and 50 *μ*L of streptavidin-HRP antibodies were incorporated. A 60-minute incubation at 37°C followed, succeeded by five washes with washing buffer. Subsequently, 50 *μ*L of substrate solutions A and B were individually introduced to each well. An additional 10-minute incubation at 37°C under dark conditions ensued. Termination involved the addition of 50 *μ*L of stop solution to each well. This treatment was executed across six replications. Within a span of less than 10 minutes, the optical density (OD) values were recorded through an ELISA reader (Thermo Scientific™ Multiskan™ GO Microplate Spectrophotometer) at a wavelength of 450 nm.

### 2.6. Caspase Levels Analysis

Caspase-3 and caspase-9 levels were assessed through the utilization of an ELISA kit (Bioassay Technology, Shanghai, China), adhering to the provided manufacturing protocol. Each well plate was allocated a total of 40 L of supernatant originating from HeLa cell cultures. Subsequent to this, mouse antibody caspase-3 was introduced for the quantification of caspase-3 levels, while mouse antibody caspase-9 was employed for the evaluation of caspase-9 levels. A sequential addition of 50 L of streptavidin-HRP followed, culminating in an 1-hour incubation at room temperature. Following this, a thrice-repeated washing step was undertaken using a washing buffer. Successively, 50 L of substrate A solution was dispensed into each well plate. Subsequently, substrate B was introduced and incubated in the dark at 37°C for a duration of 10 minutes. The conclusion of this process involved the addition of 50 L of stop solution. This treatment was executed across six replications. Measurement occurred via an ELISA reader (Thermo Scientific™ Multiskan™ GO Microplate Spectrophotometer) at a wavelength of 450 nm.

### 2.7. Apoptosis Index

The detection of apoptosis was executed through flow cytometry [35]. In 6-well plates, a total of 1 × 10^5^ HeLa cells were subjected to incubation with *G. applanatum* extract and medium under 5% CO_2_ at 37°C overnight. Subsequent to this incubation, the mixture of *G. applanatum* extract and medium was withdrawn. The addition of a solution composed of PBS, EDTA, and trypsin transpired in each well, followed by a 2-minute incubation within the CO_2_ incubator. The aspirates from each well were then combined in Eppendorf tubes and centrifuged at 2500 rpm for 5 minutes. Postcentrifugation, cells within each Eppendorf were subjected to a cold PBS wash, followed by centrifugation at 1500 rpm at 4°C. A suspension of 400 L binding buffer incorporating 5 L annexin V-FITC and 10 L PI was prepared, and a 10-minute incubation at 4°C in darkness ensued. This treatment was executed across six replications. This protocol was replicated six times. Flow cytometry analysis was undertaken using guava® Flow Cytometry easyCyte™ Systems.

### 2.8. Statistical Analysis

All data were presented as mean ± SD and subsequently subjected to analysis using GraphPad Prism software version 8 (San Diego, CA, USA). The one-way ANOVA test was employed to ascertain the significance of GACP extract impact. Following this, the Tukey test was administered to highlight significant disparities between groups. A statistical significance threshold was established at *p* < 0.05.

## 3. Results

### 3.1. FTIR Analysis

**Figure 1:**
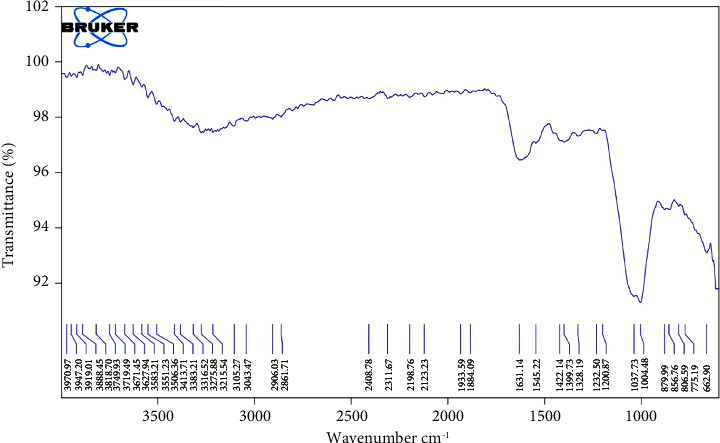
FTIR analysis of *G. applanatum* crude polysaccharide extracts.

The FTIR analysis results are depicted in Figure 1. The analysis unveiled the existence of multiple functional groups through the observation of a total of 41 peaks. Notably, a robust peak at 1631.14 cm^−1^, indicative of C=C stretching, signifies the presence of alkenes within the GACP extracts. In addition, a peak at 1399.73 cm^−1^, corresponding to C-H stretching, suggested the existence of alkanes within the GACP extracts. Moreover, a pronounced peak at 1004.48 cm^−1^, denoting the C-O bending mode, highlighted the presence of compounds such as alcohols, carboxylic acids, esters, and ethers. Furthermore, a range of peaks spanning from 3970.97 cm^−1^ to 1884.09 cm^−1^ showcased various functional groups, including alcohol (O-H stretching), amine (N-H stretching), carboxylic acid (O-H stretching), alkane (C-H stretching), alkyne (C=C stretching), and allene (C=C=C stretching).

### 3.2. Cell Viability

**Figure 2:**
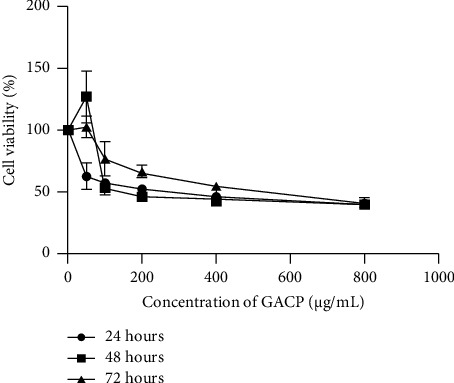
Effect of *G. applanatum* crude polysaccharide extracts on cell viability of HeLa cells. *G. applanatum* crude polysaccharide extracts with various concentrations were treated at three incubation times (24, 48, and 72 hours).

The percentage of cell viability is illustrated in Figure 2. The outcomes exhibited a reduction in cell viability with increasing doses of GACP extracts. Notably, the administration of GACP extracts for 24 hours resulted in viabilities of 62.46% at 50 *µ*g/mL, 56.60% at 100 *µ*g/mL, 51.90% at 200 *µ*g/mL, 45.51% at 400 *µ*g/mL, and 39.80% at 800 *µ*g/mL. In addition, at 48 hours, cell viability percentages were recorded as 126.99% at 50 *µ*g/mL, 53.15% at 100 *µ*g/mL, 46.07% at 200 *µ*g/mL, 43.57% at 400 *µ*g/mL, and 40.09% at 800 *µ*g/mL. Furthermore, the cell viability percentages at 72 hours demonstrated values of 102.52% at 50 *µ*g/mL, 76.52% at 100 *µ*g/mL, 66.20% at 200 *µ*g/mL, 54.86% at 400 *µ*g/mL, and 39.02% at 800 *µ*g/mL.

### 3.3. Apoptotic Index

**Figure 3:**
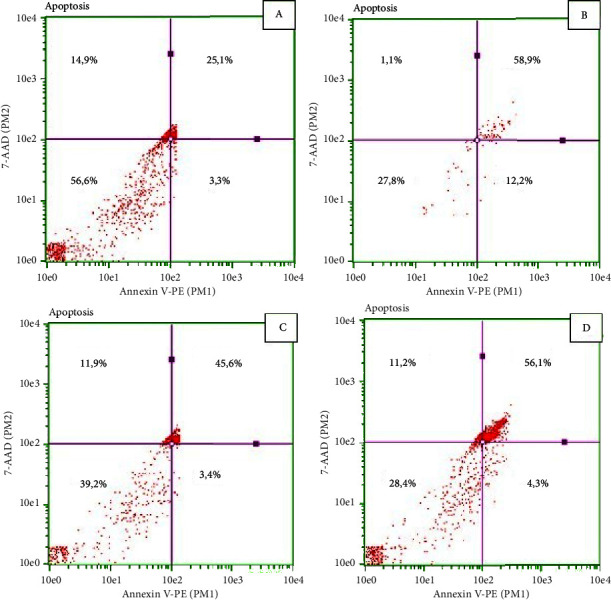
Effect of *G. applanatum* crude polysaccharide extracts on apoptotic index by flow cytometry test. (a) *K*− group; (b) *K*+ group; (c) P1 group; (d) P2 group. Bottom left: live cells; bottom right: early apoptosis; top right: late apoptosis; top left: necrosis.

The apoptotic index is illustrated in Figure 3. In the normal control group (A), live cells constituted 56.6%, early apoptosis was 3.3%, final apoptosis reached 25.1%, and necrosis accounted for 14.9%. In the positive control group (B), live cells were at 27.8%, early apoptosis was 12.2%, final apoptosis was 58.9%, and necrosis was minimal at 1.1%. For the treatment group with GACP extract 200 *µ*g/mL (C), live cells were 39.2%, early apoptosis accounted for 3.4%, final apoptosis was 45.6%, and necrosis was 11.9%. Similarly, in the treatment group with GACP extract 400 *µ*g/mL (D), live cells constituted 28.4%, early apoptosis was 4.3%, final apoptosis reached 56.1%, and necrosis was 11.2%.

### 3.4. Effect of *G. applanatum* Extract on Proinflammatory Cytokine

**Figure 4:**
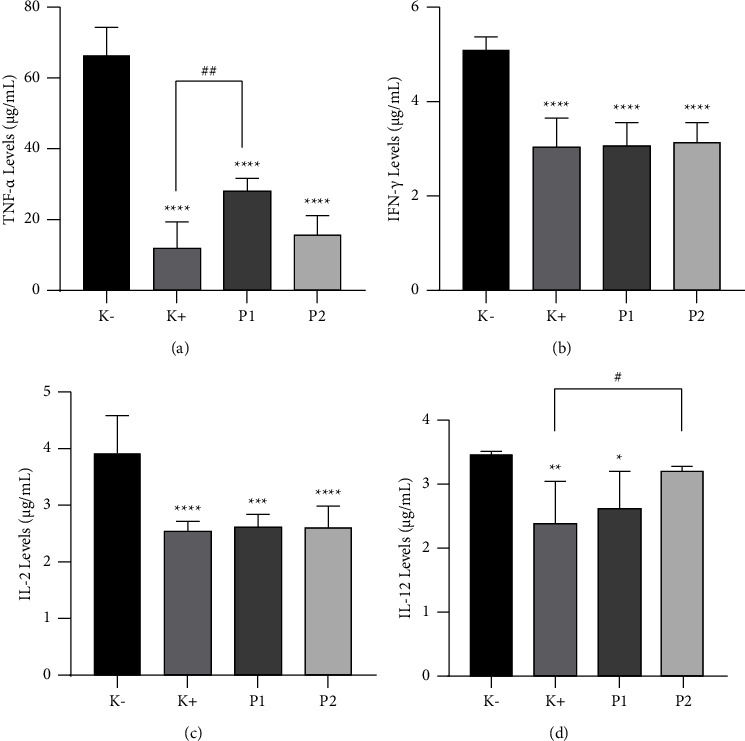
Effect of *G. applanatum* crude polysaccharide extracts on proinflammatory cytokine parameters. (a) TNF-*α* levels, (b) IFN-*γ* levels, (c) IL-2 levels, and (d) IL-12 levels. Data are presented as mean ± SD (*n* = 6). ^*∗∗∗∗*^*p* < 0.0001 compared to the negative group (*K*−). ^*∗∗∗*^*p* < 0.001 compared to the negative group (*K*−). ^*∗∗*^*p* < 0.01 compared to the negative group (*K*−). ^*∗*^*p* < 0.05 compared to the negative group (*K*−). ^##^*p* < 0.01 compared to the positive group (*K*+). ^#^*p* < 0.05 compared to the positive group (*K*+). *K*−: negative control; *K*+: positive control; P1: *G. applanatum* crude polysaccharide extract 100 *µ*g/mL; P2: *G. applanatum* crude polysaccharide extract 200 *µ*g/mL. TNF-*α*: tumor necrosis factor-*α*, IFN-*γ*: interferon-*γ*, IL-2: interleukin-2, and IL-12: interleukin-12.

Figure 4 presents the effect of *G. applanatum* extract on proinflammatory cytokine levels. Specifically, TNF-*α* levels were measured at 65.80 ± 8.25 *µ*g/mL for *K*−, 11.86 ± 7.18 *µ*g/mL for *K*+, 19.41 ± 1.42 *µ*g/mL for P1, and 15.58 ± 5.32 *µ*g/mL for P2. For IFN-*γ*, levels were 5.09 ± 0.27 *µ*g/mL for *K*−, 3.05 ± 0.60 *µ*g/mL for *K*+, 3.08 ± 0.46 *µ*g/mL for P1, and 3.16 ± 0.38 *µ*g/mL for P2. Correspondingly, IL-2 levels were found to be 3.88 ± 0.66 *µ*g/mL for *K*−, 2.54 ± 0.13 *µ*g/mL for *K*+, 2.60 ± 0.20 *µ*g/mL for P1, and 2.57 ± 0.38 *µ*g/mL for P2. In addition, IL-12 levels were 3.46 ± 0.04 *µ*g/mL for *K*−, 2.38 ± 0.63 *µ*g/mL for *K*+, 2.62 ± 0.55 *µ*g/mL for P1, and 3.21 ± 0.06 *µ*g/mL for P2. Importantly, in the positive group and GACP-treated groups, parameters such as TNF-*α* levels, IFN-*γ* levels, IL-2 levels, and IL-12 levels were significantly (*p* < 0.05) lower than those in the negative control group. The administration of GACP extract effectively mitigated the increase in TNF-*α* levels, IFN-*γ* levels, IL-2 levels, and IL-12 levels when compared to the negative control group. These results indicated that the GACP extract effectively played a protective role against cancer. Comparative analysis between the effects of doxorubicin and GACP extract on proinflammatory cytokine parameters demonstrated that doxorubicin had a lower impact than GACP extract on HeLa cells. Nevertheless, GACP extract exhibited significant effects compared to the negative control group, affirming its potential as an anticancer agent.

### 3.5. Effect of *G. applanatum* Extract on Caspase-9 and Caspase-3

**Figure 5:**
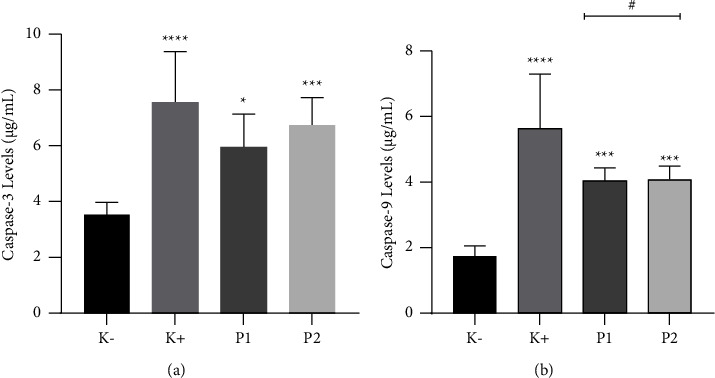
Effect of *G. applanatum* crude polysaccharide extracts on caspase-9 and caspase-3 levels. (a) Caspase-3 levels, (b) caspase-9 levels. Data are presented as mean ± SD (*n* = 6). ^*∗∗∗∗*^*p* < 0.0001 compared to the negative group (*K*−). ^*∗∗∗*^*p* < 0.001 compared to the negative group (*K*−). ^*∗*^*p* < 0.05 compared to the negative group (*K*−). ^#^*p* < 0.05 compared to the positive group (*K*+). *K*−: negative control; *K*+: positive control; P1: *G. applanatum* crude polysaccharide extract 100 *µ*g/mL; P2: *G. applanatum* crude polysaccharide extract 200 *µ*g/mL.

Figure 5 illustrates the impact of *G. applanatum* extract on caspase-9 and caspase-3 levels. Regarding caspase-3 levels, values were found to be 3.60 ± 0.42 *µ*g/mL for *K*−, 7.61 ± 1.87 *µ*g/mL for *K*+, 6.012 ± 1.173 *µ*g/mL for P1, and 6.88 ± 0.96 *µ*g/mL for P2. On the other hand, for caspase-9, the levels were 1.73 ± 0.22 *µ*g/mL for *K*−, 5.67 ± 1.62 *µ*g/mL for *K*+, 4.05 ± 0.34 *µ*g/mL for P1, and 4.105 ± 0.37 *µ*g/mL for P2. These ELISA assay results strongly indicate that GACP significantly increased caspase-9 and caspase-3 levels, implying that GACP potentially induced apoptosis through the classical nuclear DNA damage pathways. Notably, the expression of caspase-9 and caspase-3 in the GACP-treated groups and the positive control group was significantly (*p* < 0.05) higher than that in the negative control group. When comparing the effects of doxorubicin and GACP extract on caspase levels, substantial differences were observed. Doxorubicin led to higher increases in caspase-9 and caspase-3 levels than those in the GACP-treated groups. Nevertheless, the administration of GACP extract significantly increased caspase-9 and caspase-3 levels, underscoring its role in apoptosis induction and affirming its potential as an anticancer agent.

## 4. Discussion

The utilization of natural sources as a safe approach for drug development, especially in cancer treatment, has gained prominence. In this study, GACP was administered to HeLa cells to assess cell viability. The results of GACP exposure over 24, 48, and 72 hours exhibited varying percentages of cell viability. Notably, the 72-hour exposure yielded higher cell viability percentages compared to 24 and 48 hours at higher doses. This condition could be attributed to cell adaptation in HeLa cells. Extended exposure to GACP, such as at 72 hours, may have facilitated cell survival and replication. The findings suggest that GACP does not exert toxic effects on HeLa cells. Chronic conditions often lead to increased levels of proinflammatory cytokines. In the case of HeLa cells, they produce proinflammatory cytokines such as TNF-*α*, IL-6, IL-2, IL-12, and IFN-*γ* to sustain tumor development and proliferation over the long term [36, 37]. Moreover, proinflammatory cytokines can drive angiogenesis and metastasis within the tumor microenvironment [21, 38]. The activation of NF-*κ*B is a critical factor in promoting proinflammatory cytokine abundance. The prevention of NF-*κ*B activation has become a significant focus in the field of cancer treatment. In this study, GACP demonstrated the ability to decrease levels of TNF-*α*, IL-6, IL-2, IL-12, and IFN-*γ*. The polysaccharides present in GACP are thought to inhibit IK*β* phosphorylation, thereby hindering the translocation of NF-*κβ*. This mechanism ultimately results in lower proinflammatory cytokine production by HeLa cells [22, 39].

The study also explored the impact of GACP on the apoptosis pathway in HeLa cells. The decrease in proinflammatory cytokine levels contributed to a reduced survival rate of HeLa cells, thereby initiating the apoptosis process [40–42]. This study aims to examine GACP effects in apoptosis, measured by flow cytometry. The use of Annexin V-FITC allowed for the detection of early apoptosis, as it can identify phospholipid phosphatidylserine (PS) exposed on the outer plasma membrane. In addition, propidium iodide (PI) was employed to stain nuclear DNA, enabling the identification of late apoptosis and necrosis following the loss of cell membrane integrity. The dose of 400 *µ*g/mL of GACP exhibited the highest percentage of late apoptosis at 48 hours. However, the optimal dose was found to be 200 *µ*g/mL at 24 hours, as it led to 39.2% viable cells without inducing cell toxicity while still inducing apoptosis in 45.6% of HeLa cells. Notably, the percentage of early apoptosis induced by doxorubicin was higher than that induced by GACP treatment or the control group. This suggests that GACP holds potential as an anticancer agent, particularly in triggering late apoptosis. Furthermore, the study elucidated the underlying mechanism of GACP-induced apoptosis, particularly through the intrinsic pathway. The treatment with GACP led to elevated levels of caspase-9 and caspase-3 compared to the control group. Caspase-9, known as an initiator caspase, plays a pivotal role in activating executor caspases, such as caspase-3 [43–45]. Apoptosis is initiated by an increase in proapoptotic proteins like BAX, BAK1, BIM, BID, and BBC3, which stimulate mitochondria to release cytochrome c into the cytoplasm. This triggers the formation of the apoptosome and activation of caspase-3 [25]. The apoptosome forms when cytochrome c binds to Apaf-1, followed by the subsequent joining of procaspase-9 to the apoptosome, leading to its cleavage and activation. The activation of caspase-9 further stimulates the activation of caspase-3 [26], which orchestrates apoptosis by cleaving and activating proteins crucial for the proteolytic degradation of cancer cells. Ultimately, this process leads to the elimination of cancer cells and the suppression of tumor growth. The enhancement of caspase-9 and caspase-3 production suggests that GACP holds potential as an anticancer agent, particularly for the treatment of cervical cancer.

Previous studies have underscored the potential therapeutic effects of *G. applanatum* crude extract in cervical cancer treatment. These effects include inducing apoptosis via the intrinsic pathway, inhibiting DNA survival and transcription factor proliferation, causing DNA fragmentation, and curbing the growth and metastatic potential of cervical cancer cells [46]. The existing literature on *G. applanatum*'s anticancer properties further solidifies its potential in cancer therapy, including its toxicity against tumor cells compared to normal cells [47].

## 5. Conclusion

In conclusion, the polysaccharide compounds obtained from *G. applanatum* have the capability to induce apoptosis in HeLa cells by upregulating the levels of caspase-3 and caspase-9. However, to gain a deeper understanding of the underlying molecular mechanisms driven by *G. applanatum* extract, further investigations are warranted. Further research should prioritize the analysis of protein interactions, protein signaling, and regulatory factors implicated in apoptotic responses, particularly through animal studies (*in vivo*).

## Data Availability

The data used to support the study are included within the article.

## Abbreviations

PBS: Phosphate-buffered saline
EDTA: Ethylenediaminetetraacetic acid
BAX: Bcl-2-associated X protein
BCL-2: B-cell lymphoma 2
BAK 1: Bcl-2 homologous antagonist killer
BIM: Bcl-2-interacting mediator of cell death
BID: BH3-interacting domain death agonist
BBC3: Bcl-2 binding component 3
ELISA: Enzyme-linked immunosorbent assay
DMEM: Dulbecco's modified Eagle medium
TNF-*α*: Tumor necrosis factor-alpha
IFN-*γ*: Interferon-gamma
IL-2: Interleukin-2
IL-6: Interleukin-6
IL-12: Interleukin-12
FTIR: Fourier transform infrared
NF-*κ*B: Nuclear factor kappa B
Apaf-1: Apoptotic protease activating factor-1.
